# The Mental Health Consequences of Coronavirus Disease 2019 Pandemic in Dentistry

**DOI:** 10.1017/dmp.2020.190

**Published:** 2020-06-05

**Authors:** Andrea Vergara-Buenaventura, Mariella Chavez-Tuñon, Carmen Castro-Ruiz

**Affiliations:** Department of Periodontology, School of Dentistry, Universidad Cientifica del Sur, Lima, Peru

**Keywords:** anxiety, coronavirus, COVID-19, health personnel, public health dentistry

## Abstract

With the spread of coronavirus disease 2019 (COVID-19), strict isolation strategies to limit virus transmission have been applied worldwide. The lockdown has affected and challenged different medical areas. Doctors, nurses, dentists, and other health care workers are concerned about contagion, not only for themselves, but also for their families and colleagues. Furthermore, the oral mucosa has been accepted as a high-risk route of transmission for COVID-19. In many countries, dentists have been forced to stop working during quarantine until further notification. Isolation and its financial impact have produced physical and psychological pressure, depression, social anxiety, and other mental health concerns. This article aims to provide a comprehensive review of the consequences of past epidemics on mental health and to assess possible aspects that might be associated with mental implications in dentists during the COVID-19 pandemic. Finally, some concrete actions to avoid subsequent potential consequences are recommended.

Since the World Health Organization (WHO) declared coronavirus disease 2019 (COVID-19) a pandemic, it became a major challenging public health problem worldwide.^1,2^ Many governments have declared a quarantine and focused on reducing transmission by limiting activities to save lives but, at the same time, impacting the economy of people.^3,4^ This context has generated anxiety, uncertainty, and fear in people and among health care workers (HCW).^5,6^

The mental impact of the COVID-19 epidemic on patients, children,^7^ older adults,^8^ and students^9^ has been reported. However, little attention has been paid to psychological well-being and burnout between HWC,^5,10^ especially between dental practitioners.

This article aims to provide a comprehensive review of the consequences of past epidemics on mental health and to assess possible aspects that might be associated with mental implications in dentists during the COVID-19 pandemic. Finally, some concrete actions to avoid subsequent potential consequences are recommended.

## IMPACT OF PAST EPIDEMICS ON MENTAL HEALTH

It is essential to consider the impact of past epidemics on psychological well-being. Data from the last epidemics showed that quarantined patients developed psychiatric symptoms. Due to its high mortality and infection rate, the severe acute respiratory syndrome (SARS) epidemic caused anxiety and panic in the affected countries,^11^ and patients infected with Middle East respiratory syndrome coronavirus (MERS-CoV) had a significant impact on their mental health during quarantine.^12^

COVID-19 was initially detected in 2019 in Wuhan city, Hubei Province, China.^13^ The rapid increase of contagions and fast-spreading virus caused the WHO to raise the disease to pandemic status in March 2020.^2^ This quarantine has produced many stressful life events: the loss of freedom, separation from relatives and loved ones, and the inability to work has harmed mental health.^4,14^

## IMPACT OF COVID-19 PANDEMIC ON THE DENTIST’S LIFE

General measures have been established for all, including HCWs, but, in addition to social distancing and mobility restriction measures, HCWs have to wear stricter personal protective equipment (PPE) at work.^3,15^ Regrettably, with the lack of PPE for health care personnel, COVID-19 has revealed epidemiological and governmental deficiencies to prevent the spread of the disease.^16^

The COVID-19 outbreak has negatively impacted the activity of dentists.^17^ Routine dental procedures have been suspended because of the risk of cross-infection during dental care.^18,19^ Moreover, oral mucosa has been recognized as an entry route of infection, limiting dental activities to treat urgent and emergency procedures to minimize the production of drops or sprays.^20^ Additionally, dental companies and industries have decided to suspend part of their staff.^21^ Dentists feel a moral duty to reduce regular work to avoid spreading the infection among their relatives and patients. However, they also have a great concern about the financial consequences of a lockdown.^10,22^ Consolo et al.^17^ reported that almost 85% of dentists in a province of Italy reported being worried about contracting the infection during dental procedures. Shacham et al.^23^ identified psychological distress among dentists and found that the fear of getting infected with COVID-19 from a patient provides high psychological tension. On the other hand, a high number of patients have been canceling their previous appointments,^17^ and others have perceived vulnerability to infection during dental practice.^24^

A point often overlooked is the lack of inclusion of dental personnel in the public health emergency management system. In most countries, dentists do not participate in the direct care of patients infected with COVID-19.^10^ It has been reported that medical staff unable to work in clinical areas or not posted directly on the front lines may feel guilty.^25^

Meanwhile, the suspension of educational institutions has also affected dental education.^26^ Dental students have been required to stay at home and learn online during this pandemic.^18^ However, it has been previously reported that the suspension of educational and research activities can lead to frustration.^27^

## SIGNS AND SYMPTOMS

Previous studies have described the effects of isolation on mental health in HCWs.^4,14^ Acute stress reactions included physical, cognitive, social, and emotional reactions or a combination of these.^28^ Lee et al.^29^ have reported irritability, low mood, insomnia, and posttraumatic stress symptoms among residents and hospital staff after quarantine. Burnout can be shown as a reduced sense of accomplishment, emotional exhaustion, or depersonalization. These phenomena have a direct negative impact on anxiety, stress, fatigue, mood disorders, depression, poor patient quality care, and, in worst cases, substance abuse and suicides.^14,30^ During the SARS epidemic, being in contact with infected patients was a predictor of developing anxiety, exhaustion, poor concentration, detachment from others, and deteriorating work performance.^31^

During this pandemic, medical workers have been dealing with the risk of infection, isolation from their families, discrimination, and overworking with inadequate protection, developing frustration, and exhaustion.^32^ Fear of the unknown can cause anxiety to develop in healthy people as well as in those with previous mental health problems.^33^ In dentists, the appearance of psychological symptoms has been related to the fear of contracting COVID-19, especially when having a background disease or a higher subjective overload.^23^

## STRATEGIES AND RECOMMENDATIONS

Well-being is not only the absence of emotional distress, but also it implies a positive mental, social, and physical condition.^34^ Self-care and psychological flexibility are essential aspects of psychological health.^35^ Experts recommend being self-compassionate, staying active, and having time for leisure activities.^6,36^ Additionally, they encourage healthy sleeping, eating, drinking, and exercising, and trying to focus on the positives.^28^

The importance of maintaining physical activity is well understood. There is a risk that a vulnerable sector of the population falls short of staying active, affecting immunity that directly influences the probability of infection.^37^

Taking care of ourselves, keeping in touch with family and friends, and finding sources of inspiration and joy are strongly recommended.^6,36^ Social media can assist in communication with relatives, allowing isolated people to receive updates about the situation of loved ones.^14^

Some protocols have been established for doctors but not for dentists. Many health professionals working in health institutions are not qualified to provide mental health assistance during pandemics.^38^ In hospitals and dental clinics, surveys should be used to measure the physical, emotional, and mental exhaustion of the staff, and counseling services should be established using digital technologies.^38^ According to Torales et al.,^32^ mental health support should be provided even at 6 months after the end of isolation in people with vulnerable mental health.

Remote psychological support should be implemented. Daily free text messages, phone calls, and video calls with supportive tips may be performed to support the mental health of those in isolation or lockdown.^28,39^ The creation of programs has been suggested to provide economic support during quarantine to those families with lower incomes and those whose family members cannot work.^40^ On the other hand, it is time to see this situation as a forced opportunity to review and restructure our businesses.^21^ Consider proactive approaches at home to avoid financial loss and avoid boredom.^40^ An alternative approach to maintain the dentist-patient relationship is by using the Internet along with technology such as apps or software to facilitate communication via chat, telemedicine, and/or videoconference.^15,41^

## CONCLUSION

Dentistry has to be part of the health care system. Dentists must be prepared to play a more critical role in the public health emergency management system and to fight against emerging life-threatening diseases.^42,43^ Finally, yet importantly, some renowned institutions like the American Dental Association have prepared online courses to provide an overview of the return to work after COVID-19 quarantine.^44^

Although isolation has demonstrated it’s effectiveness in reducing the spread of disease, depriving people of their liberty needs to be handled carefully. Supporting the mental health of all HCWs must be a critical part of the public health response, and special efforts should be directed to vulnerable sectors. Dentists must take measures to make this experience, which is not yet over, as tolerable as possible, and public health officials while taking action must be mindful of sectors that seem to be forgotten.
